# Bag-based rapid and safe seed-train expansion method for *Trichoplusia ni* suspension cells

**DOI:** 10.1186/1753-6561-5-S8-P124

**Published:** 2011-11-22

**Authors:** Nicole C Bögli, Christoph Ries, Irina Bauer, Thorsten Adams, Gerhard Greller, Regine Eibl, Dieter Eibl

**Affiliations:** 1 Institute of Biotechnology, Zurich University of Applied Sciences and Facility Management (ZHAW), Wädenswil CH-8820, Switzerland; 2 Sartorius Stedim Biotech, Göttingen, D-37075, Germany

## 

*Trichoplusia ni* suspension cells (High Five™) used in conjunction with the baculovirus vector expression system (BEVS) are regarded as potential product system of new, recombinant virus-like particle (VLP) vaccines. In order to push vaccine development and production, biomanufacturers use single-use technology when- and wherever possible. This applies to upstream processing and in particular seed-train expansion ranging from cryopreserved vials via t-flasks, spinners (respectively shake flasks) to stirred stainless steel bioreactors. The stainless steel bioreactors deliver inoculum for seed bioreactors and have been increasingly replaced by wave-mixed single-use bag bioreactors during the last 5 years [1].

The approach presented for seed-train cell expansion of High Five suspension cells is based on the Biostat CultiBag RM50 optical (Sartorius Stedim Biotech). It was used for the production of cells for long-term storage and for the expansion of cells for subsequent production experiments. For long-term storage the cells were frozen at high cell concentrations (20 - 40 x 10^6^ cells x mL^-1^) in 60 mL Cryobags and stored in nitrogen at -196 °C in vapour phase.

Initial experiments were aimed at the growth characterization of High Five suspension cells from a vial working cell bank (WCB). The High Five cells were grown in batch mode and in 250 mL single-use shake flasks (Corning and Sartorius Stedim Biotech) on a Certomat® CT Plus shaker (Sartorius Stedim Biotech) during six days (triplicates, 27 °C, 100 rpm, 25 mm shaking diameter). Afterwards a procedure was developed in which thawed cells from a Cryobag were directly transferred into and expanded in a Biostat CultiBag RM. Under optimal process conditions (500 mL starting volume, a starting cell density of 1 x 10^6^ cells x mL^-1^, 27 °C, rocking angle of 6 °, 20 - 30 rpm, 0.2 vvm, DO set point 50%) growth rate (0.039 - 0.042 h^-1^), doubling time (18 - 20 h) and maximal cell density (7.8 - 8.9 x 10^6^ cells x mL^-1^) showed good correlation with results arising from CultiBags which were inoculated with cells from shake flasks. This bag-based seed-train expansion allows time saving of about one week and reduces cross-contamination, both advantages being due to omitted intermediate cultivation steps in shake flasks.
